# The multicultural conceptualisation of well-being: The role of culture and circumstance in operationalising well-being in South Africa: A mixed-methods approach

**DOI:** 10.1186/s12889-023-16966-0

**Published:** 2023-10-19

**Authors:** Adekunle Adedeji, Tosin Tunrayo Olonisakin, Johanna Buchcik, Franka Metzner, Wandile Tsabedze, Klaus Boehnke, Erhabor S. Idemudia

**Affiliations:** 1 Faculty of Life Sciences, Hamburg University of Applied Sciences, Hamburg, Germany; 2 Bremen International Graduate School of Social Sciences (BIGSSS), Constructor University, Bremen, Germany; 3 Faculty of Humanities, North West University, Mafikeng, South Africa; 4 Department of Psychology and Behavioural Studies, Ekiti State University, Ado-Ekiti, Nigeria; 5 Center for Psychosocial Medicine, Department of Medical Psychology, University Medical Center, Hamburg-Eppendorf, Hamburg, Germany

**Keywords:** Well-being, Multicultural setting, Socioeconomic status, Racial inequality, Quality of life

## Abstract

**Background:**

Exploring and understanding indicators of better life outcomes have remained popular among social and health researchers. However, the subjective approach to measuring well-being has raised questions on the appropriateness of standard measures of well-being in multicultural settings. The current study examines generalised well-being and its dependence on the implicit understanding of individual culture and circumstances.

**Methods:**

A mixed-method approach with a cross-sectional design and focus group discussions was adopted. Fifteen focus group discussions with 66 participants were conducted in four provinces of South Africa. Descriptive statistics, correlations, regression analysis and analysis of variance were computed for quantitative data. The focus group discussions were analysed using a content analysis approach. The recorded focus group discussions were transcribed using the intelligent verbatim technique. Data analysis was done stepwise using open, axial, and selective coding techniques.

**Results:**

Quantitative analysis showed a strong and significant association between quality of life and income and a moderate association with educational attainment. The open coding technique for qualitative data confirmed 11 different subconstructs of well-being, mentioned 403 times during the 15 focus group discussions. Furthermore, well-being indicators varied based on participants’ racial identity.

**Conclusions:**

The findings confirm personal circumstance and culture as significant for interpreting results from well-being research. Furthermore, it supports Maslow’s Hierarchy of Needs, highlighting the movement from deficiency needs to growth needs after deficiency needs are met. Research must adopt a more sociological approach to improve the accuracy and implementibility of findings when using standardised measures of well-being.

## Background

Understanding and measuring well-being and its differentiation between cultures based on demographic characteristics have remained central in health and social research [1–3]. However, a lack of a clear definition and a broadly accepted operationalisation of this construct remain a challenge. This challenge is explained in different well-being discourses: a dynamic concept that includes subjectivity, social and psychological dimensions and health-related behaviours. The consensus is that well-being considers both a subjective and an objective dimension [4] and is a multifaceted construct closely related to health.

Scientific discourse has often referred to the World Health Organization (WHO) definition of health as “a state of complete physical, mental and social well-being and not merely the absence of disease or infirmity” [5]. However, well-being – originally from the emotional and psychological domains of health [6], is often used interchangeably with quality of life, life satisfaction or health-related quality of life [3, 7]. This makes a consensus about a precise definition complex and almost impossible.

Knight and McNaught showcase this broad complexity of well-being in a framework that incorporates aspects of individual well-being, family well-being, community well-being, and societal well-being [8]. Although this framework brings some transparency to the well-being construct and allows researchers and practitioners to develop and plan health-related and health-promoting interventions, it remains multidimensional and complex to operationalise. Another effort to simplify well-being in a “Background paper by WHO secretariat for developing a comprehensive mental health action plan” [9] connects well-being and health. Here, well-being was assumed to be central to overcoming daily tasks like forming relationships, studying, working, or pursuing leisure interests. Individual attributes and behaviours, environmental factors, and social and economic circumstances were identified as contributing factors to well-being. These factors interact, are dynamic, and can change individuals’ subjective well-being.

### Subjectivity and measures of well-being

Accessing well-being from a subjective perspective means considering an individual’s self-evaluation concerning happiness and life satisfaction [3] as well as mental health problems, vulnerabilities, and risks [9]. Today, it is accepted that the construct of well-being can be considered from an individual point of view using self-report measures. This acknowledges the variability of well-being aspects such as health, income, relationships or support from family members on the individual level [10].

Subjective well-being can be measured using qualitative or quantitative methods. Qualitative approaches use guided face-to-face interviews and focus groups or observation methods to collect personal views on influential factors in people’s lives (e.g., spirituality and religion), which are rarely quantified [11]. Over the years, various research projects have been conducted to develop and standardise quantitative well-being measures. Cooke et al. [12] systematically searched databases for self-report instruments assessing well-being. They identified 42 instruments that varied significantly in length, psychometric properties, and their conceptualisations and operationalisations of well-being. They found a wide divergence across the different theoretical conceptualisations of well-being and how well-being is operationalised within particular theoretical categories. One of the examined scales is the Satisfaction With Life Scale (SWLS) [13]. This five-item scale was developed and validated to measure global life satisfaction and subjective well-being between different age and gender groups. The scale measures subjective well-being’s cognitive component among the general population.

Another brief scale is the WHO-5 Well-being Index. The 5-item instrument classifies responses between scores of poorest well-being and best possible well-being. It includes items on feeling cheerful, calm, relaxed, active, vigorous, fresh, rested, and interested [14]. The WHO measure has been primarily used among patient groups to rank individual subjective well-being [15–17].

The Ryff Scales of Psychological Well-being (PWB) is comprehensive and available either as a long version with 84 questions or a shorter version with 54, 42, and 18 questions. It includes self-acceptance, positive relations, a sense of autonomy, managing the environment, purpose in life, and personal growth [18, 19]. The PWB Scale has been operationalised among American adults of all ages, including lower-income backgrounds [18, 20]. Similarly, the PWB scale has been used among Latino college students [21], African-Americans and Mexican-Americans [22].

Generally, it is clear that these measures vary in the dimension of well-being they measure and how they measure it. These variabilities in well-being measures emphasise that individual attributes, culture, and behaviours might be crucial to well-being, including how people deal with thoughts and feelings and manage their daily lives, behave to improve their health, and participate in daily social activities.

Only a few of these measures of well-being were developed and tested in multicultural samples, including non-Western cultures outside Europe, Australia or the United States (US). Keyes et al. [23] evaluated the 14-item Mental Health Continuum–Short Form (MHC–SF) in a random sample of 1,050 Setswana-speaking adults in the North-West province of South Africa. They replicated the three-factor structure of emotional, psychological and social well-being found in US samples. Santos et al. [24] conducted the adaptation and cross-cultural validation of the Brazilian version of the 14-item Warwick-Edinburgh Mental Well-Being Scale in a multi-stage process covering translation, back translation, cognitive interviews, expert evaluation, pre-test and applying the final version of the instrument to a sample of 122 college students. Fen et al. [25] developed and validated the 30-item Singapore Mental Well-being Scale based on interviews, surveys and focus group discussions in Asian multi-ethnic samples of Chinese, Indian and Malaysian adults. All these measures identified culturally unique determinants of well-being in the different study samples.

### Culture and well-being

Well-being in a cross-cultural context has continued to gain attention in social discourse. Nowok and colleagues, for example, examined if migration directly affects the well-being of migrants. They found that migrants are happier directly after migration but that this advantage and their subjective well-being decline at other times due to a lack of opportunities [26]. Similarly, Cutrona et al. [27] revealed a set of interrelations among community characteristics, individual characteristics, and psychological well-being among 709 African-American women. They conclude that individuals’ and communities’ daily living and culture are connected with well-being. Another study among the different racial and cultural groups in South Africa identified different determinants of subjective well-being for the various groups [28]. The financial situation (circumstance) of Coloured and Black South Africans determines their satisfaction. At the same time, social relationships played a significant role in Asian/Indian and White participants’ subjective well-being. For all groups, subjective well-being was affected by the expectations of their future [28] and their culture.

Similarly, poverty and deprivation (e.g., a lack of access to water, sanitation, crime and violence) were identified as possible explanations for well-being in low-income countries. Several household characteristics and circumstances, including housing, sanitation, education and transportation, explain the low well-being of Black South Africans [29]. Besides social deprivation, studies have identified the experience of racism and social and cultural identity, as well as subjective integration, as factors that predict the well-being of African-Americans [30, 31] and that of African migrants in Germany [32, 33]. On the one hand, the studies presented show that perceptions and operationalisations of well-being can differ across ethnicities and cultures. On the other hand, ethnically and culturally diverse samples were often studied with measurements of well-being that were not developed for multicultural samples. A lack of well-being measures that can be used in multicultural settings based on their development with multicultural samples, especially from non-Western countries, was identified.

### The current study

Despite the complexity around measuring and operationalising subjective well-being, its effect on the experience and evaluation of life and life events, for example, migration, individual and community characteristics, socioeconomic status, and racism, remains unquestionable. Furthermore, the clear connection to health emphasises its importance for health policies to improve well-being. While research on the operationalisation of well-being is ongoing, several models have offered a categorisation of well-being’s multidimensionality [8]. Despite these efforts and partial successes, the search for accurate and flexible measures of well-being remains crucial for the successful implementation of public health policies. The current study contributes to this search by exploring the role of individual circumstance, cultural identity, and connectivity through group formation and memberships in conceptualising what makes them feel well. For this, we identify South Africa as a multiracial and multicultural setting that allows for exploring the complexity of subjective well-being.

South Africa is known for its multicultural and multiracial characteristic. It is often referred to as a rainbow nation regarding culture [34], language [35] and traditional practices [36]. The diverse cultural identities have historically influenced the structural and systemic designs of social, economic and political spheres [37]. This is partly attributed to the racial categorisation and segregation that emerged in apartheid South Africa. The racial categories of White, Black, Indian, and Coloured that emerged in apartheid South Africa has remained the social identifier for the country’s population groups. This grouping has since then shaped South African’s lived experiences and life outcomes and made race relations an essential determinant of well-being. [29, 38]. South Africa’s population consists of 76.4% Black South Africans, 9.1% White South Africans, 8.9% Coloured South Africans, 2.5% Indian South Africans, and 0.5% from other or unspecified racial backgrounds [39]. Despite this racial diversity, South Africa is widely recognised for its significant social and economic inequality and is considered one of the most unequal nations globally [40]. This inequality along racial lines curves individual circumstances, cultural identity, and interaction with other cultures [41].

### Research goal and objectives

The current study explores well-being as an individual’s subjective evaluation of their lives in relation to their understanding of self, collectiveness, culture, and circumstances. This definition acknowledges the variability of well-being over a lifetime and its interpretation within a life-course approach [9]. The multidimensional characteristics of well-being emphasise the importance of cultural, collective and circumstantial understanding of the construct of well-being and its role in promoting overall life quality. The current study explores the difference in the subjective conceptualisation of well-being among four major South African racial groups. We examine subjective well-being and its dependence on the implicit understanding of the self, culture, and circumstances. In doing so, we:

1. identify determinants of well-being for the four racial groups, that is, Black, Coloured, White and Indian South Africans,
2. highlight the unique determinants of well-being for each of the aforementioned racial groups, and.
3. explore collective determinants of well-being irrespective of racial identity.

## Methods

### Study design

This study used a mixed-methods approach. The quantitative approach used standardised instruments to collect data on participants’ quality of life, socioeconomic status, and demographic features. The qualitative approach utilised content analysis to explore the participants’ understanding and experience of well-being. The approach was used because race relations in South Africa is an emotionally charged domain and racial identity shapes social reality for individuals. Participants of different racial groups could express their experiences and opinions of how their racial identity feautures in their well-being. Fifteen focus group discussion (FGDs) were conducted in four provinces in South Africa. The FGDs allow participants to express and interpret the world around them in the context of their sociocultural environment [42]. This method has been noted as having the flexibility and adaptability to suit subjective evaluations of life and experience [43, 44]. Furthermore, it allows for an in-depth exploration, identification and interpretation of themes in social research.

### Sampling and sample/participants

**Fig. 1:**
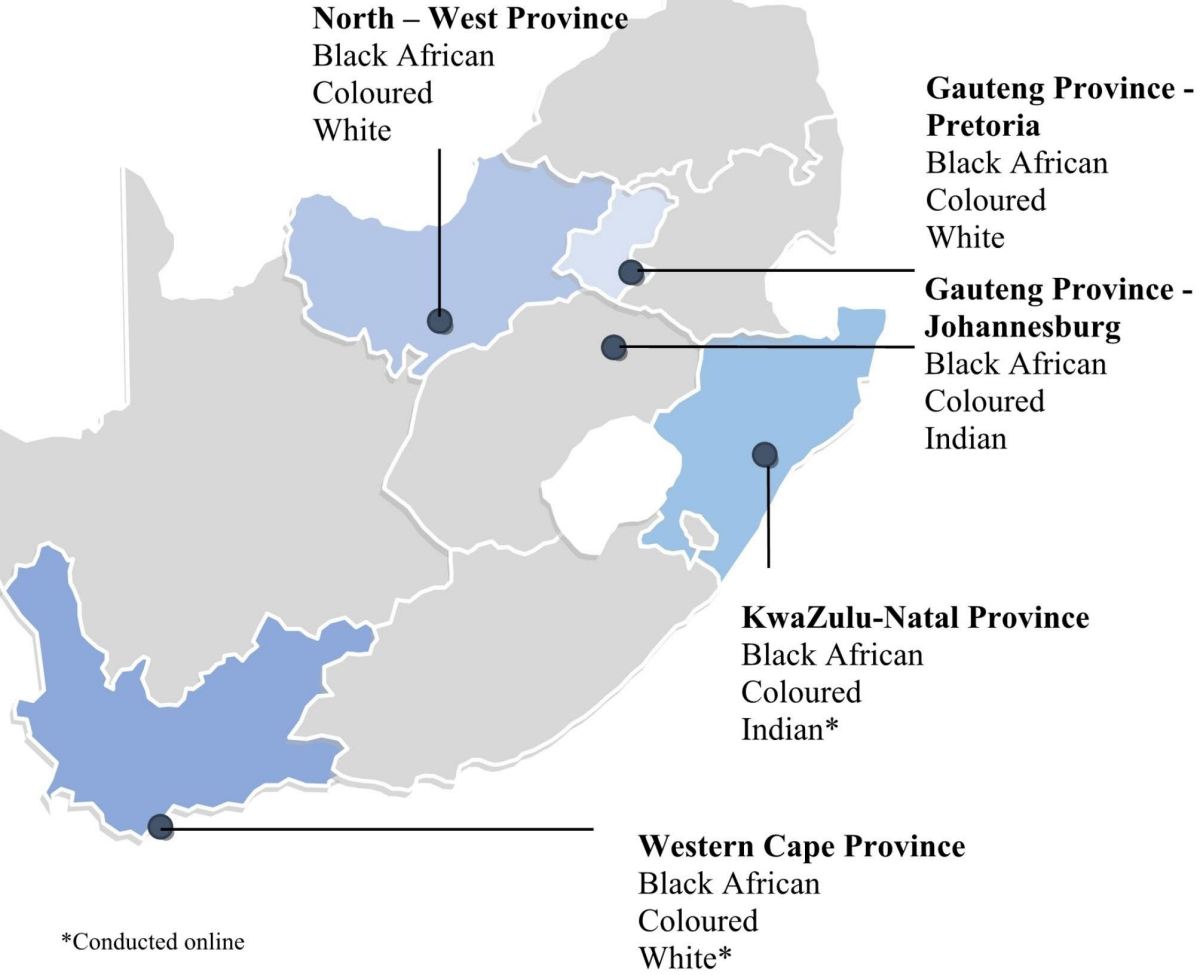
Location for focus group discussions by the province of residence in South Africa and participants’ racial identity

Four of the nine provinces in South Africa were selected for the FGDs: Gauteng, North-West, KwaZulu-Natal and Western Cape. This selection was based on the geographical positioning (i.e., North, West, South and East) and the unique demographic characteristics such as population density and the racial composite of these provinces. The Gauteng province is the most populous in South Africa and houses the country’s economic capital, Johannesburg, and the administrative capital, Pretoria. We conducted six FGDs, three in Johannesburg and three in Pretoria, to capture the complexity and diversity that characterise the Gauteng province. Three FGDs were conducted in the other provinces, that is, North-West, KwaZulu-Natal, and Western Cape. The decision on which race to invite for the FGDs was based on each selected province’s racial representation. The racial group with less than 2% representation was excluded as a rule. The included racial groups for each province are presented in Fig. 1.

Participants were recruited using a convenience sampling technique - a non-probability sampling method. Recruitment information was disseminated through personal contacts, project websites, flyers, and social media (e.g., Facebook and WhatsApp). Purposive sampling was used to ensure the participants were from various demographic categories (see Tables 1 and 2). In total, 66 South African adults (aged 20 to 71, M_age_ = 35.7 years, SD = 13.0) participated in the FGDs and completed the questionnaire. Half of the participants were females (*n* = 33), whereas 32 were males, and one reported “other” gender category. Furthermore, Black South Africans represent 39.4% (*n* = 26), Coloured South Africans 31.8% (*n* = 21), White South Africans 21.2% (*n* = 14) and Indian South Africans 7.6% (*n* = 5). More than half of the participants were from Gauteng (33%) and North-West (33%) provinces. About 18% were from KwaZulu-Natal, whereas 15.2% were from the Western Cape.

**Table 1:** Sample demographic characteristics (*n* = 66)

		*N*	%
Gender	Female	33	50.0
	Male	32	48.5
	Others	1	1.5
Racial Identity	Black South African	26	39.4
	Coloured South African	21	31.8
	White South African	14	21.2
	Indian South African	5	7.6

**Table 2:** Sample socioeconomic characteristics’ frequency distribution (*n *= 66)

		Total *(n)*	Black *(n)*	Coloured *(n)*	White *(n)*	Indian *(n)*	*F*	*p*
Educational attainment	Did not complete primary school	1	–	1	–	–	0.48	0.79
	Completed Primary school	1	–	1	–	–		
	Some secondary	7	3	4	–	–		
	Matric or equivalent	17	5	8	2	2		
	Tertiary education	39	17	7	12	3		
	Doctorate/postdoctoral	1	1	–	–	–		
Total annual household income	Poor	20	11	6	2	1	3.86	**0.01**
	Low emerging middle class	15	8	6	–	1		
	Emerging middle class	15	5	6	2	2		
	Realised middle class	8	1	3	4	–		
	Upper middle class	8	1	–	6	1		
	Emerging affluent	–	–	–	–	–		
Occupation in relation to qualification	No occupation at present	27	10	12	3	2	1.72	0.17
	Below my qualification level	15	9	2	4	–		
	At my qualification level	21	6	5	7	3		
	Above my qualification level	3	1	2	–	–		

### The quantitative measure

A structured questionnaire was administered to the participants before the interview. Each participant was provided with a printed questionnaire and a pencil. Upon completion, the questionnaire was piled together with other study documents to maintain anonymity. Sociodemographic and socioeconomic data were collected regarding age, racial identity, gender, marital status, level of education, occupation, and income. Age was measured as the number of years lived. Similarly, participants were asked to choose which racial group they identify with from five options, that is, Black, White, Coloured, Indian or Another racial group. Education was assessed as the highest educational level, with options ranging from none to doctorate/postdoctorate [45]. Furthermore, participants were asked to rank how well their educational attainment matches their current occupation with four options ranging from “I am not exercising an occupation at present” to “I am occupied above my qualification level”.

Household income was calculated in terms of the family’s approximate annual household income before taxes and other deductions [46]. Income was measured in South African Rand (R). Participants with yearly income below R 54,344 were coded as “poor”. Participants with income between R 54,345 and R 151,727 as “low emerging middle class”, R 151,728 to R 363,930 were coded as “emerging middle class”, R 363,931 to R 631,120 as “realised middle class”, R 631.121 to R 863,906 as “upper middle class” and R 863,906 to R 1 329,844 as “emerging affluent” [46].

In addition, a self-report questionnaire on quality of life using the EUROHISQOL 8-Items [47] was administered. The EUROHISQOL 8-item Index is a self-report questionnaire derived from the World Health Organization Quality of Life Assessment (WHOQOL-100 and WHOQOL-BREF instruments) [48]. It includes eight items representing the physical, psychological, social, and environmental quality of life. The eight items were scored on a 5-point Likert scale ranging from “not at all” to “completely”. The overall QoL score was computed as the eight items’ aggregate scores ranging from 8 to 40, with higher scores indicating better QoL. The questionnaire presented good reliability in the current sample with a Cronbach’s alpha value of 0.87.

### Focus group discussions about the conceptualisation of well-being

The fifteen FGDs with 66 participants were held between June and December 2021. There was an average of five participants per discussion. The FGDs were conducted in four provinces and eight cities: North-West (Mafikeng, Klerksdorp, Potchefstroom), Gauteng (Johannesburg and Pretoria), KwaZulu Natal (Durban), and Western Cape (Cape Town). The FGDs lasted an average of 1 h 45 min. On arrival, participants received an information leaflet and were required to complete a consent form. Before starting the discussion, members of the research team were introduced to the participants. Equally, all participants introduced themselves. This helped create a relaxed atmosphere for the participants to ease their entry into the research. Furthermore, the participants were informed of the ground rules guiding the discussion and that it would be recorded. Given that complete anonymity could not be guaranteed with FGDs, participants were assured that personally identifying information about them will not be published.

To capture the individual subjective interpretation of well-being, each participant was asked to describe features, things, or significant events in everyday life that make them feel well. In addition, participants were provided with a paper to list those things essential for their well-being. This paper was collected at the end of the FDGs to collate points raised during the discussion. Furthermore, follow-up questions were asked to evaluate participants’ access to those things participants outlined as facilitators of well-being. For example, “How satisfied are you with the availability of those things compared to someone of a different race, gender, or age”. To encourage conversation flow and involve other participants in the discussions, follow-up questions were asked, for instance, “Does someone (i.e., another participant) share this feeling or view” and “How is that for you?”. The follow-up questions ensured the participants could interact and express themselves freely [49].

### Data analysis

#### Quantitative data analysis

Descriptive statistics were computed for sociodemographics, socioeconomic status and quality of life. Bivariate correlations r, stepwise multiple regressions, and analyses of variance (ANOVA) were computed using SPSS28. Effect sizes were interpreted as small (*r*/ß = 0.10), medium (*r*/ß = 0.30) or large (*r*/ß = 0.50) [50].

#### Qualitative data analysis

The focus group discussions were analysed using a content analysis. The recorded focus groups discussions were transcribed using the intelligent verbatim technique. Data analysis was performed stepwise using the open, axial, and selective coding technique [51]. First, open coding was used to identify determinants of subjective well-being. Then, axial and selective coding was used to interpret and explain the racial differences in the conceptualisation of well-being. These codes consisted of short sentences or single words, for example, ‘Occupation’ (i.e., in socioeconomic subconstruct) and ‘Friendship’ (i.e., in social relationship subconstruct). Two researchers (AA and TO) independently coded and analysed each transcript using Atlas.ti.

## Results

A descriptive analysis of the participants’ socioeconomic features shows that about 60% reported having at least a tertiary education. The data suggested that about 41% were not formally employed at the time of the FGDs. Nearly 30% were poor based on annual income (Table 2). Descriptive data analysis on quality of life shows a mean score of M = 27.56 (SD 6.47) for the total sample (*n* = 66). Results from the group-specific analysis indicate that Coloured South African participants (*n* = 21) reported the lowest mean score, M = 26.19 (SD = 6.17), followed by Black South Africans M = 26.31 (SD = 6.82) (*n* = 26). White South Africans (*n* = 14) reported the second highest mean score M = 29.63 (SD = 5.43), whereas the Indian South African (*n* = 5) participants reported the highest mean M = 34.00 (SD = 4.00).

Further analyses of the racial differences in socioeconomic status using ANOVA show significant racial disparities in income but no statistical difference in educational attainment and occupation.

### Quantitative analysis – bivariate analysis and ANOVA

A Pearson correlation coefficient matrix was computed to examine the relationship between quality of life (EUROHISQOL 8-item), age, income, and educational attainment for the 66 participants in the FGDs. Quality of life showed a strong, significant positive association with income (*r* = .54, *p *< .01) and a moderate association with educational attainment (*r *= .34, *p *< .01). No significant association was established between age and quality of life. Further analysis comparing the quality of life mean score for the different racial groups showed no significant variation in the means based on racial identity. A stepwise multiple regression using all mentioned sociodemographic variables as predictors—categorical variables (racial identity and marital status) contrast-recoded into dichotomous variables—confirms this finding: Only income (ß = 0.42, p < .01) and educational attainment (ß = 0.24, p = .04) significantly predict the quality of life with a medium to high effect size.

### Qualitative analysis – indicators of well-being

**Fig. 2:**
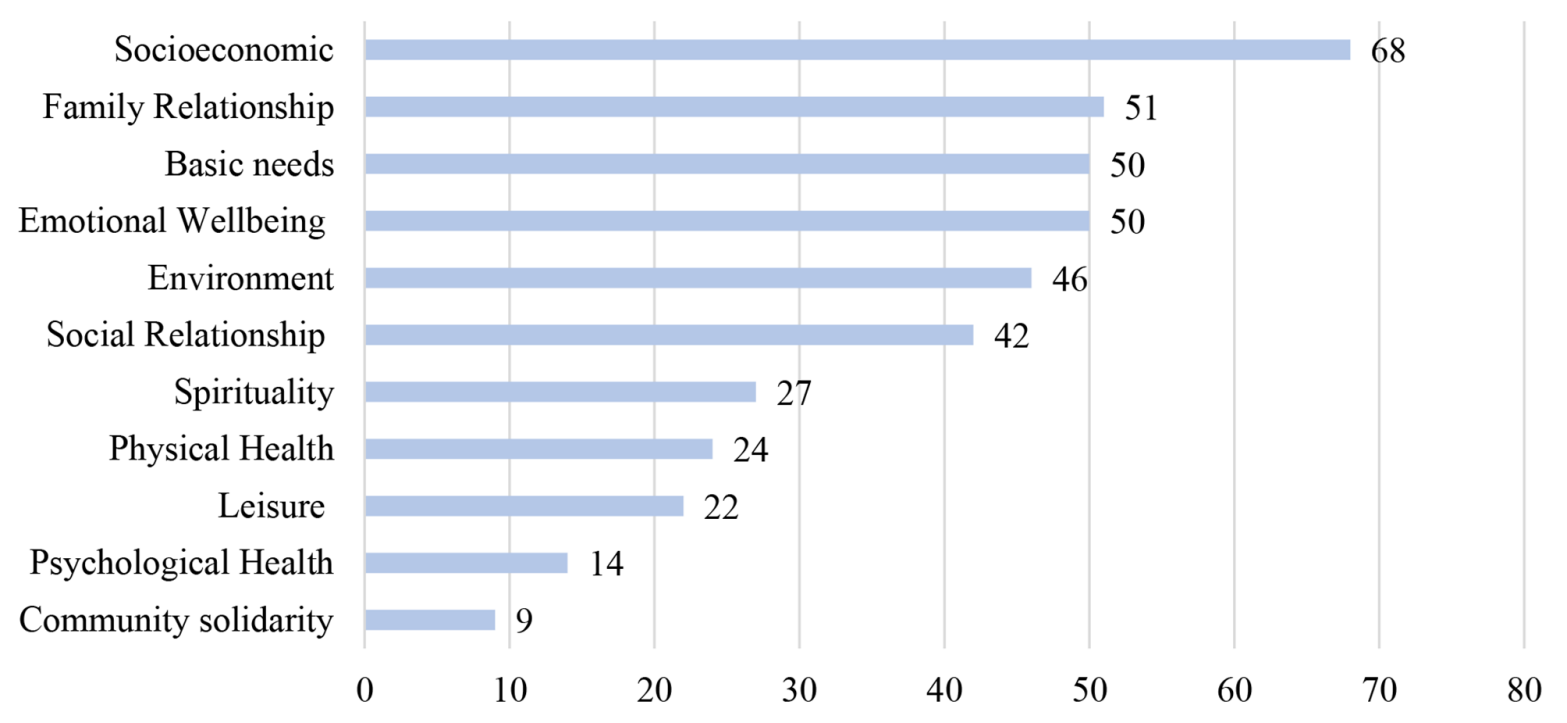
Subconstructs of well-being by categories and how often they were mentioned during the 15 focus group discussions (n = 66)

Results from open, axial, and selective coding techniques identified 11 subconstructs of well-being among the different racial groups in South Africa. Participants’ subjective well-being was understood as physical health, psychological health, emotional health, social relationship, family, spirituality, basic needs, leisure, community solidarity, environment, and socioeconomic status. The open coding technique confirmed that the different subconstructs of well-being were mentioned 403 times during the 15 FGDs by the 66 participants (see Fig. 2). Pseudonyms were used to report the participants’ comments.

#### Physical health

The physical health subconstruct of well-being is conceptualised as the ability to maintain a healthy life that allows the performance of day-to-day activities without unnecessary tiredness or physical stress. Twenty-four of the 403 mentions of well-being indicators were coded and categorised as the physical health subconstruct. This includes caring for the body and recognising habits and behaviours that significantly impact overall health, *GJIND01*: *“… I must feel that I’ve put time into my appearance. My health is very important, so I try and eat healthily and drink water… to feel well”*. Participants in the FGDs emphasised that positive physical health in terms of biological functioning facilitates better life while poor health negatively affects well-being; for example, *WCBLK01: “…knowing that I’m healthy and able to perform my daily task… makes me feel well” or GJIND02: “…my health and energy levels. It is essential in my everyday life, because if I’m not feeling well, or if my energy levels are low, then I can’t perform daily tasks.”.*

#### Psychological health

Psychological health constituted 3.5% of the mentioned indicators of well-being. Psychological health, in terms of mental health, determines how a person handles stress and makes choices. In the current sample, psychological health was confirmed as an aspect of well-being among South Africans. For example, *GPBLK01* mentioned that *“…maintaining my mental health, in addition to physical health is important for my well-being”.* Similarly, for *GJBLK01: “I feel well mostly when … also when I’m able to take care of my mental health”*, and *NWCMR04: “… mental health is very important to avoid physical problems because the mental problem can cause more personal problems.”*

#### Emotional well-being

The ability to produce positive emotions, feelings, thoughts and cope with discomfort and stressful situations constitute emotional well-being and represents 12.4% of the coded mentions of well-being. Feeling of happiness – for example, described by *NWWHT06: “…getting strength through motivation, positivity or happiness …makes me feel well”*, sense of belonging, creative satisfaction, self-improvement, future aspirations, love – *GPBLK02: “I feel well mostly in a safe environment when I feel loved”*, emotional support and motivation were highlighted as features of emotional well-being in the current sample.

#### Spirituality

Following Joseph et al. (2017 p 506), spirituality refers to “a more general, unstructured, personalised, and naturally occurring phenomenon, where a person seeks closeness and/or connectedness between him/herself and a higher power or purpose.” Participants in the FGDs highlighted their relationship with God and their association with religious institutions as crucial to their well-being. Twenty-seven out of the 403 mentions of well-being indicators emphasised spirituality, e.g. by *NWBLK09*: *“For me, God is the most important thing for me to feel well”, WCBLK02: “…and also my relationship with God is very essential in my everyday life. I cannot go out of the house without having to pray, or you know, talk just talking to God in general.”* and by *GPWHT04: “… connecting with God. So these are things I’ve tried to do, but I don’t achieve them every day, to pray to God and walk with Him… achieving these makes me feel well.”.*

#### Social relationships

Social relationships refer to individual interactions and relationships in terms of friendship support that reoccur and are perceived by the participants to have personal meaning. About 10% of the mentions of various well-being indicators were categorised under social relationships. This includes general positive relationships - as formulated by *NWBLK04: “…I can add that positive social relationship make me feel well.”*, friendships *- GPCMR01: “…having good relations among my peers is one of the good things to have.”* and social interactions as addressed by *WCCMR02: “Things that make me feel well will be something just like a social interaction with people…”.*

#### Family relationship

Similar to social relationships, interactions and relationships with family with personal meaning were also highlighted as contributory to well-being among the current sample. These constituted 12.7% of the coded indicators of well-being and the second most repeated indicators. These indicators include spousal relationship and support, for example, raised by *NWWHT02: “…my husband is a big part of my future as well. He helps me to actually look forward to the future and want to build myself up, not just emotionally.”* as well as general family relationship and support, mentioned for example by *NWWHT04: “My family is number one really makes me feel good about life. I have a very supportive and nurturing family. My parents are still together. And I feel like we are my brother, we get along great, and that makes me feel well.”.*

#### Leisure

Participating in leisure activities was also important in improving subjective well-being. Participants across the different racial groups highlighted participating in activities such as reading, watching a movie, singing - *NWBLK02: “What makes … having a nice time with my household of course and singing”*, walking the dog, shopping or cooking facilitates better well-being - *GPWHT01*: *“So what’s important for me to be happy every day is to relax. I take time every day to relax and destress and consciously let go of my worries. I like to escape. So every day, I do something like watch a criminal crime movie”*. About 5.5% of coded indicators were categorised as a leisure subconstruct of well-being.

#### Basic needs

Human basic needs are crucial for survival. Meeting these basic needs – like having food and water, safety, and shelter – was highlighted as a predictor of well-being among the South African sample that participated in the FGDs. This is how *GPBLK05* described it: *“For me, I will feel well if I have a sense of security, to shelter….” WCBLK02 stated: “…what is essential in my everyday life is if I don’t have food, I get so worried, so the fridge needs to be stacked up”*, and *WCCMR* specified: *“Good food to conduct life. Food gives me energy that makes you feel well.”*. About 12.4% of coded indicators fall under the basic needs subconstruct of well-being.

#### Community solidarity

Community solidarity includes beneficial behaviour that facilitates better communal outcomes [52]. Community action such as providing assistance, collaboration, and community service were highlighted as determinants of well-being among the South African sample and accounted for 2.2% of the coded indicators of well-being, as addressed by *NWWHT02*: “*I’m excited to go to work because I help my communities.”, NWCMR06: “The gratitude I get from helping people and knowing I’m helping people in my community makes me feel well”*, and *GPWHT01: “Community collectiveness is very important for my well-being because it provides security and safety in my neighbourhood”.*

#### Environment

Environmental determinants of well-being include exterior agents that can be causally linked to a change in health or well-being status. For the current study, factors such as the experience of discrimination, stereotype, functional government, access to information - *GJBLK04*: *“I think it’s the responsibility of the government to make sure that the information is accessible. Having this information is important to make me feel well”*, and access to infrastructure, among many others, were identified as important determinants of well-being. *GJBLK03* stated: *“…all the environments directly prevent … from accessing whatever you want. …even the educational system is not accessible. The ability and the potential are there, there is no access”. KZNCMR 02* mentioned: *“…the experience of racial stereotypes prevents me from accessing my need ….”*. About 11.4% of coded measures were categorised as an environmental subconstruct of well-being.

#### Socioeconomic status

Having enough money, a stable job, and advancement in education and training were also highlighted as essential determinants of well-being among the study sample. For example, *KZNCMR04* formulated: *“And for my family, I need to have a substantial amount of money in the bank at a specific time. We need medical aid. We also need fuel in our cars.”. GJIND02* described it like this: *“Also, the thing you mentioned, money gives me some peace of mind knowing that I have something. Because I feel like the problems are there. And it’s easier when money isn’t the problem because we have so many problems,”* and *NWBLK01* stated: *“I feel well when I’m working, for getting money to do everything I want in my life.”.* The socioeconomic subconstruct of well-being was the most mentioned indicator. It accounted for 16.9% of the mentioned indicators of well-being during the 15 FGDs.

### Racial differences in well-being measures

**Fig. 3:**
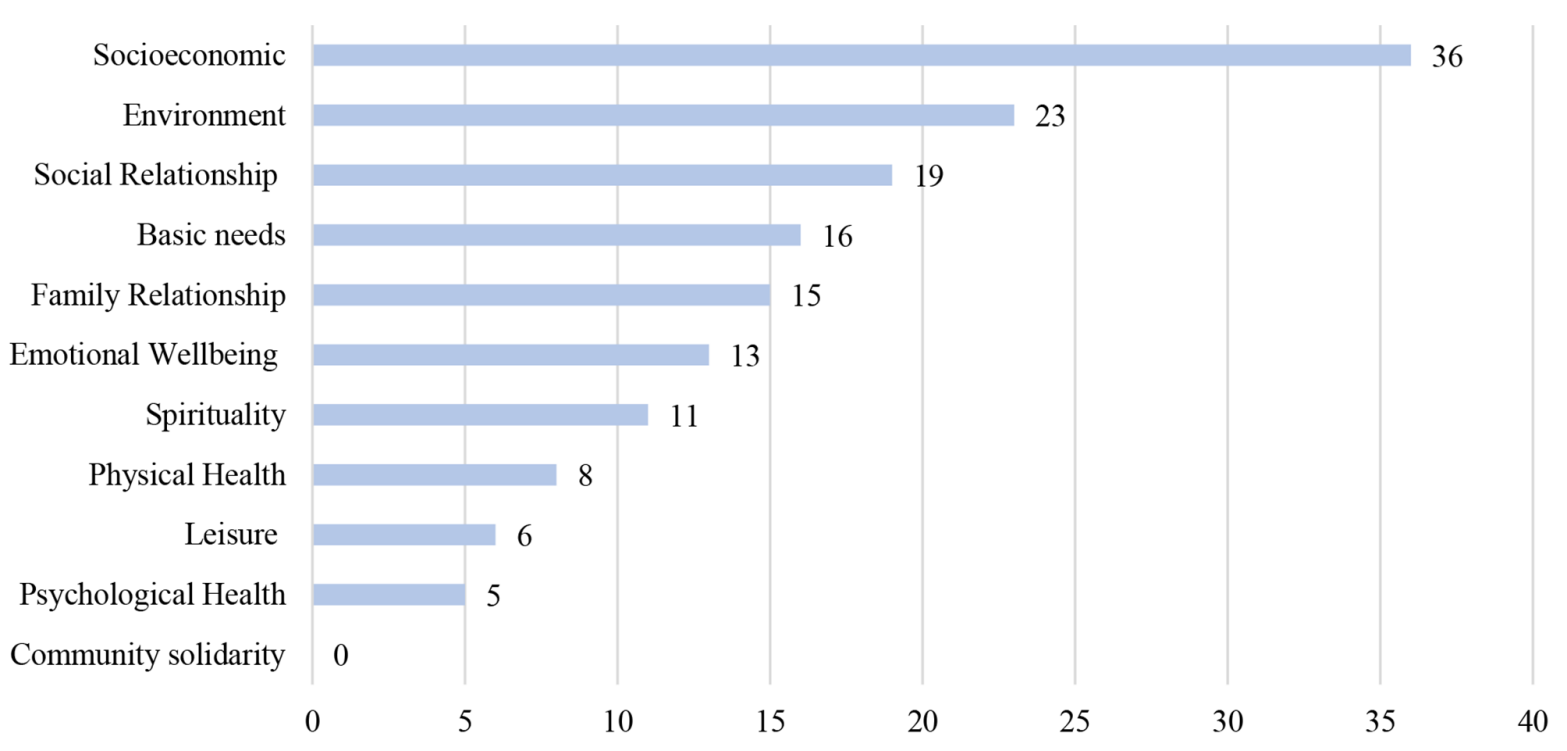
Frequency of coded subconstructs of well-being by racial groups – Black South Africans (*n* = 26)

**Fig. 4:**
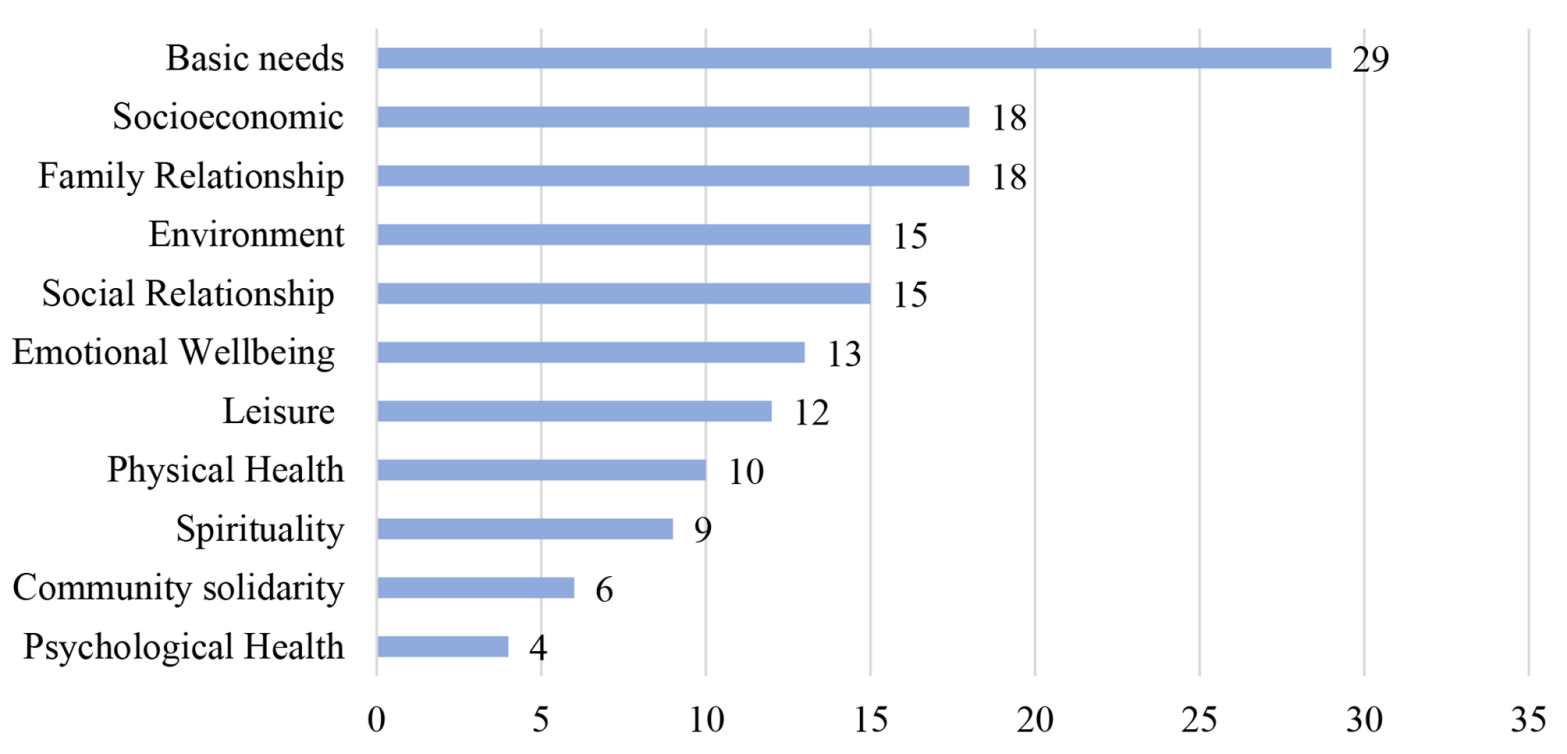
Frequency of coded subconstructs of well-being by racial groups – Coloured South African (*n* = 21)

**Fig. 5:**
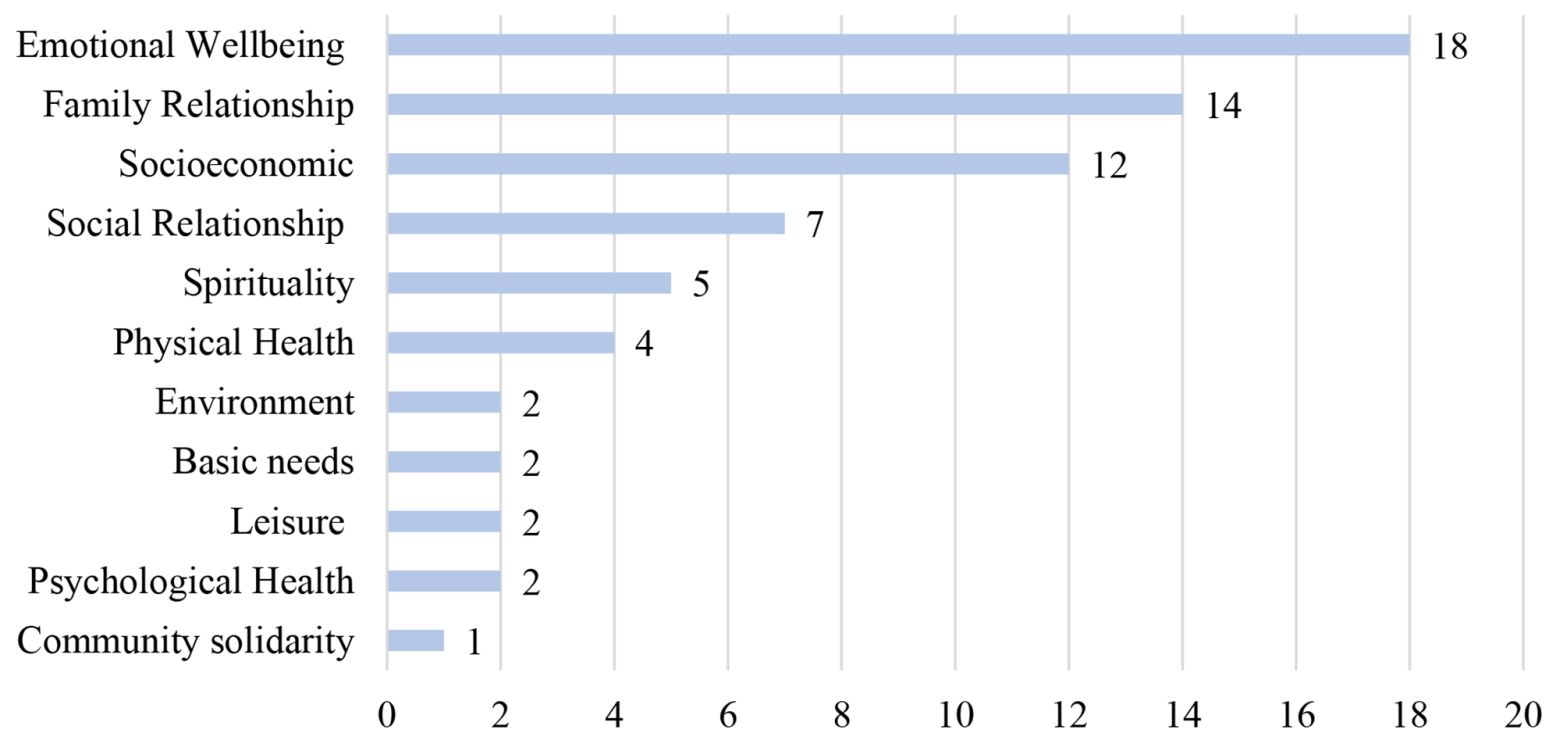
Frequency of coded subconstructs of well-being by racial groups - White South African (*n* = 14)

**Fig. 6:**
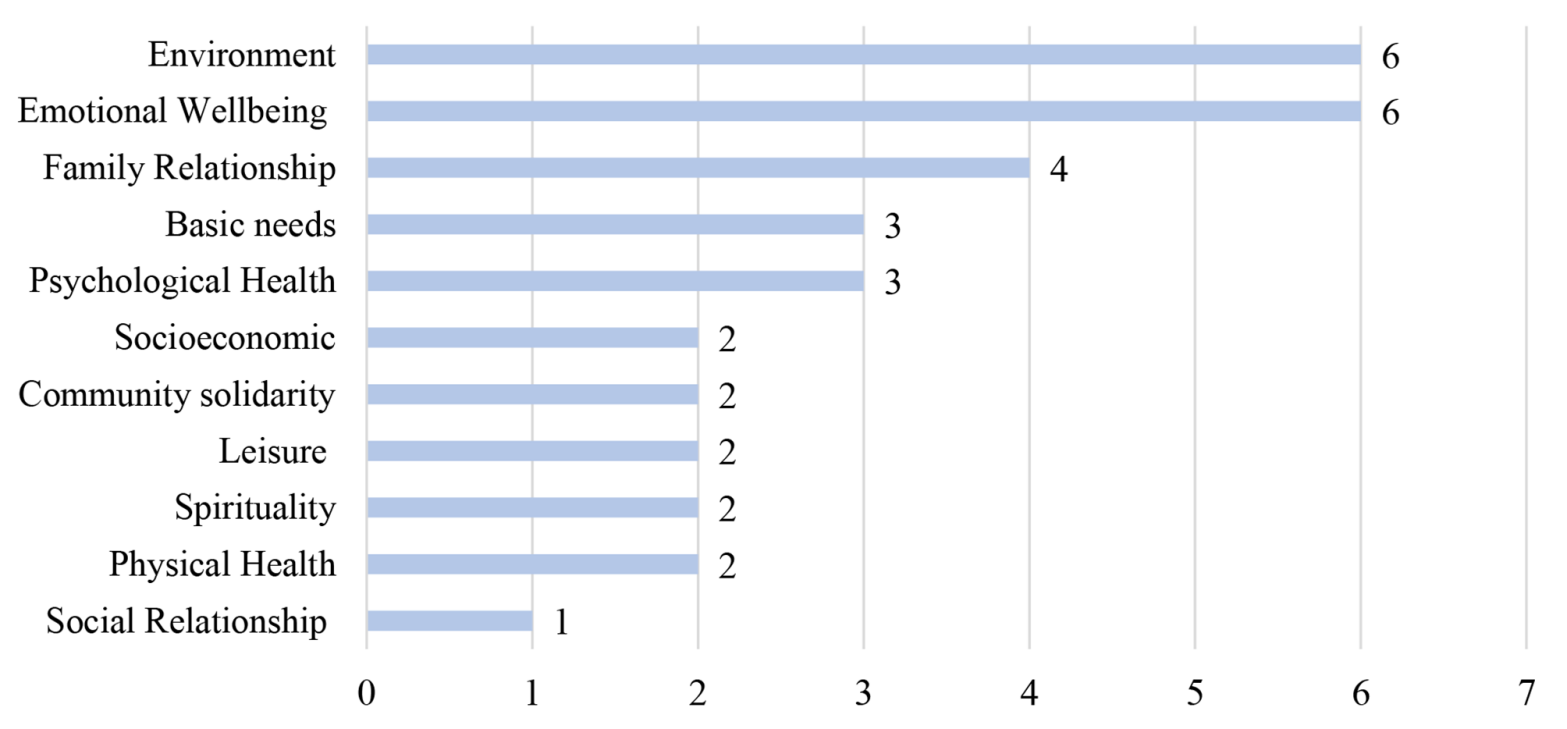
Frequency of coded subconstructs of well-being by racial groups - well-being Indian racial groups (*n *= 5)

The results from the open, axial and selective coding to explore and explain the racial differences in the conceptualised well-being show unique and common determinants for all racial groups (see Figs. 3, 4, 5 and 6).

### Black South African group

For the 26 Black South Africans that took part in five FGDs across South Africa, all but the community solidarity subconstruct were mentioned as an important determinant of well-being. However, socioeconomic status (24%), environmental domain (15%), social relationship (12.5%), basic needs (10.5%), and family relationship (9.9%) constitute 72% of the total mentioned indicators of well-being.

Furthermore, the participants projected socioeconomic status as the most important indicator of well-being. They further expound on how socioeconomic status is linked to other indicators of well-being, for example, how having an income is related to better physical health, a safer environment and better psychological health. *GPBLK04*: *“… people’s priorities are mostly according to their racial groups … Black people are mostly looking for employment, safety, education… White South Africans have the privilege and care more about happiness or fulfilment. For us Black people, we don’t have the privilege to start caring about our happiness; it’s survival first.”.*

Similarly, the environmental domain including government functionality, access to infrastructures, social discrimination and stereotypes was also highlighted as a necessary and multifaceted determinant of well-being. For example, *NWBLK02* stated: *“… looking at the statistics of people who have medical aids in South Africa, more Whites, and Indians, compared to their Black have accessibility to medical aid. Also, the kind of healthcare system Black people are exposed to, White South Africans rarely would go to a local clinic because they have more resources to go to private hospitals and meet their medical needs. So, as a young professional, I find medical aid expensive … it’s not like I don´t want medical aids. It’s just too expensive or inaccessible, and I’d rather redirect that money to something else like sending money back home”.*

### Coloured South African group

Twenty-one Coloured South Africans participated in five FGDs. For Coloured South Africans, all 11 coded subconstructs of well-being were mentioned as important determinants of well-being. Basic needs (19.5%), socioeconomic status (12.1%), family relationship (12.1%), environment (10.1%), social relationships (10.1%), emotional well-being (8.7%) represent about 73%- of all mentioned indicators of well-being. Meeting basic needs and a better socioeconomic status represent more than 30%. *GJCMR04* described: *“…safety and decent living space, food, clean water, to see the kids happy to be able to provide for them and my parents …these things are important to many Coloured”.* Similar to the Black South Africans, the determinants of well-being are multifaceted. The availability of money and other socioeconomic features seem to facilitate other determinants of well-being. For example, according to *KZNCMR05: “As coloured, it’s easier to achieve all of the things that money can’t buy that makes me feel well …so having money makes everything else easier.”.*

The environmental domain of well-being in terms of social inclusion was also emphasised, for example, by *GPCMR02: “… Coloured people are mostly belittled and locked out by other race… most other race usually associate Coloured people with drugs and other negative stereotypes, like, we are not capable of working in stores. … like if you have a Coloured surname, they won’t hire you.”.* Similarly, the environmental factors were also linked with socioeconomic status, for example, access to jobs and how it facilitates basic needs. *KZNCMR03 stated: “In the apartheid era, we [Coloured] were not white enough. Now we are not Black enough. Our children are out there; they’re not getting jobs…. Today, if you are Black, you got a better opportunity to get a job. I will tell them Coloured, and Whites are not getting jobs. Because we’re not Black enough.”.*

### White South African group

The conceptualisation of well-being among White South African participants (n = 14) in three FGDs covers all 11 coded subconstructs of well-being. However, the results show that emotional well-being (26%), family relationships (20%), socioeconomic status (17%), social relationships (10%) and spirituality (7%) accounted for 80% of the mentioned indicators of well-being. Emotional well-being in the form of happiness, love, and future aspiration was the most mentioned determinant of well-being, as stated by *GPWHT04: “It’s hard to be happy in a country where like 80% of the people are starving, and it’s terrible. And the infrastructure is crumbling. But yes, I suppose you can say that White people are better off in South Africa”* and *GPWHT01: “For me, central thing in my day is that everything ran smoothly or well sorted out.”*

For the White participants,a positive family relationship was also highlighted as an important determinant of well-being. *GPWHT01* formulated: *“… coming home to my wife and family makes me feel well”. NWWHT04* mentioned: *“My family is number one really makes me feel good about life…”*. Similarly, socioeconomic factors were also noted, as addressed by *GPWHT01: “For me, I see like two red lines that prevent well-being. On the one hand, those who are poor get discounts. They don’t have to pay for lots of things that we have to pay for. On the other hand, we have to pay for car insurance, for petrol, we have to pay for extra security, we’re actually paying more for electricity. So I think there are two facets here.”.* Furthermore, individual socioeconomic status (SES) was linked to environmental factors like governmental responsiveness and safety, for example, described by *GPWHT01: “People are being disadvantaged for a number of reasons, political decisions, or business reasons….so it’s a combination of a lot of things …*”, and by *WCWHT01: “…you’re, it’s not safe in the township [where mostly Black people live]…. For us [Whites], in the suburbs, we knew we needed to do something about our safety, so we started with what we called vigil, where people actually volunteer and say, I’m going to do this, I’m going to stay awake from eight to 12… so it’s a collective effort”.*

### Indian South African group

Five Indian South Africans participated in the FGDs in two provinces. Measures covering all 11 coded subconstructs of well-being were mentioned during the discussions. Environmental domain (18%) and emotional well-being (18%), family relationship (12%), psychological health (9%) and basic needs (9%) accounted for 66% of all mentioned indicators of well-being. Environmental domain touching on racial exclusion and government responsiveness and emotional well-being were highlighted as important and complex predictors of well-being for the Indian South African participants. *KZNIND01* stated: *“… it’s not easy to be afraid. If the person keeps quiet, nobody will care. So, the exclusion of Indians in this country is frightening … So I feel much better when I feel included”. GJIND02* formulated:*“…the government should look after all the citizens and not just certain groups”.* Similarly, family relationships and caring also stood out as an important indicator of well-being, as described by *GJIND02: “the most important thing for me is the well-being of my three children and their safety. We [Indians] are very family-oriented”.*

## Discussion

The mixed-method study aimed to explore cultural differences in the subjective conceptualisation of well-being. It further examined generalised well-being and its dependence on the implicit understanding of individual circumstances, culture, and collectivity among four major South African racial groups. Compared to White and Indian South Africans, Black and Coloured participants reported lower Socioeconomic Status (SES) in terms of education, income and working below qualification. The interracial comparison of SES was statistically significant for the participants’ income.

Quality of life significantly positively correlated with income (strongly) and education (moderately) in the total sample. Through the evaluation of the FGDs, we could find a variation in the conceptualisation of well-being based on the racial identity of the participants. In the subsample of Black South Africans, SES (e.g. money, jobs), environment (government responsiveness), social relationships, basic needs (e.g. food, water, shelter and safety), and family had the highest frequency of mentioning. For Coloured participants, basic needs, SES, family relationships, social relationships, environment and emotional well-being (e.g. love and happiness) were in the foreground of well-being. White participants most often named emotional relationships, family relationships, SES, social relationships, and spirituality as important indicators of well-being. Indian participants most often referred to environmental determinants (e.g. inclusion), emotional well-being, family relationships, psychological health and basic needs. Similarities between the racial groups were evident. SES and its related factors (e.g. basic needs) were emphasised as a constant indicator of well-being for all racial groups. Access to resources was consistently appointed as relevant to well-being. Nevertheless, across the group of participants in this study, SES was the foremost contributor to well-being among the Black participants.

The approach adopted in this study highlights the importance of circumstances in individual and group conceptualisation of well-being. Only through the additional use of focus group discussions could the subjective nature of well-being and its multidimensionality [53, 54] be corroborated. The variation in indicators of well-being reported in the current study arguably results from the different economic positions of the various racial groups [55] and cultural values. The Black and Coloured South African groups reported lower income. This low SES, characterised by low income and high unemployment, is attributed to the historical segregation of Black and Coloured South Africans [56, 57]. It is recognised that historically, White South Africans have generally experienced economic advantages due to the legacy of apartheid and its impact on resource allocation [58–60]. Therefore, the unmet socioeconomic and basic needs of Black and Coloured populations amplify the importance of SES as an indicator of well-being for these groups. The results from the FGDs show that participants from groups with lower SES ascribed their poor SES status to their unmet daily needs, insecurity, and access to medical and other social infrastructure. These findings are consistent with results from other studies where lower SES shows a stronger association with the sample’s quality of life [61] and life satisfaction [62–64].

Similarly, White South African participants’ emphasis on emotional health and family relationships mirrors findings from other studies where the participants with middle-upper social class highlighted that needs shifted from basic needs to positive emotions and feelings and relationships with family as significant predictors of happiness. These findings are also supported by Maslow’s Hierarchy of Needs [65]. White participants’ higher SES status suggested that this group has moved from deficiency needs to growth needs. According to Maslow (1987, p.69), “When a deficit need has been more or less satisfied, it will go away, and our activities become habitually directed towards meeting the next set of needs that we have yet to satisfy”. Recent statistics have shown White and Indian South Africans as the socioeconomically advantaged groups in South Africa [58–60]; therefore, satisfying growth needs generates a stronger sense of well-being for these groups.

It is clear that the mentioned indicators of well-being are interlinked. Similarly, individual circumstances are still largely dependent on racial identity and play a role in how well-being is defined and operationalised by people in our South African sample.

### Limitations and strengths

The mixed-methods approach does not allow inferring causal links between sociodemographic characteristics and well-being, respectively. Whereas the results of the qualitative focus groups allow an in-depth exploration of the topic, statements on correlations can be derived from the quantitative survey on the well-being of the sample and subsamples. When interpreting the identified associations and the similarities and differences between the racial groups, it is also important to keep in mind that race is a social construct. Racial or cultural groupings are not based on actual or ascribed biological or cultural characteristics. Still, they can be used to turn people into racialised “others” and exclude them from access to economic, social, and cultural capital, legitimising these exclusions [66]. Furthermore, it is important to note that racial self-identification holds profound implications that extend across various dimensions of life and can impact an individual’s sense of belonging, self-esteem, and overall well-being [67]. Similarly, the rating of determinants of well-being treated the racial groups as homogeneous. However, the small size and non-representativeness of the sample limit the generalizability of the results. It can be assumed that the participants within the different racial groups are a heterogeneous composition of people, despite their self-assigned group membership. At the same time, it must be recognised that the society in South Africa is characterised by diverse racial and cultural identities partly attributed to racial segregation during apartheid. For the study of a multicultural conceptualisation of well-being presented here, we identified South Africa as a representative multiracial and multicultural setting for exploring the complexity of subjective well-being. Another sampling-related limitation of this study is the underrepresentation of the Indian racial group. Notably, a major reason for this is that some of the Indian South Africans who initially indicated their interest in the study through the online advertisements later expressed their distrust for the neighbourhood in which the FGD was held. When the participants were given the option of choosing a suitable location, this did not ease their discomfort with meeting with the research team. Nevertheless, various demographic characteristics such as education, occupation, and income levels were represented in the sample of this study. Due to the cluster sampling conducted in the present study, the sample examined reflects to some extent the racial groups represented in the South African population, given the non-purposive nature of the sample.

The FGD approach adopted in this study helped gain much insight into the determinants of well-being for South Africans. This approach provided a secure setting and atmosphere for participants to express how well they experience their daily lives and the factors responsible for this. The discussion mode allowed participants to share specific experiences of how their encounters with racial discrimination hinder their well-being. Being in the midst of others with a shared social reality helped participants open up about their own experiences. This, therefore, makes the FGD approach utilised in this study appropriate for achieving the aim of this study.

## Conclusion

This study contributes to an understanding of racial and cultural disparities in various aspects of well-being. The findings of this study pinpoint the different indicators of well-being in South Africa and how racial identity influences the extent and type of well-being experienced by the citizens. The importance of socioeconomic factors in the well-being of Black and Coloured participants, for example, corresponds with current indicators that show that these groups experience economic hardship and the recurrent advocacy to improve the socioeconomic conditions of this group.

Beyond the racial group-specific and racial-group-general indicators of well-being, the participants expressed a deep interest and appreciation for a study on well-being in South Africa. Furthermore, most participants recognise that inter-group hostility and distrust engendered by colonialism and apartheid continue to create and maintain physical and social divisions between racial groups in South Africa.

This is a factor associated with the wellness of the South African population. For them, racial consciousness and racial differentiation are prominent aspects of the public life of South Africans, for example, in the workplace and other public spaces. Participants expressed interest in having an FGD that will include people of different races. They believed such a session would help understand distrust and hostility for racial outgroups from the perspective of all constituent groups. This indicates that South Africans of all racial groups believe or value dialogue as a veritable tool to facilitate the reconciliation of their differences. Perhaps this approach can be harnessed by stakeholders committed to the well-being of South Africans.

The multidimensional indicators of well-being that emerged in this study demonstrate that it is a variable that can only be understood within the socio-cultural contexts that shape individual and group experiences in South Africa. This also suggests that while there might be universal factors that contribute to the well-being of human beings, there might also be country-specific and group-specific factors that drive the experiences of wellness. However, despite the clear difference in the important dimensions of well-being for the different racial groups, the overlapping of dimensions between the groups, emphasis the similarity in experiences on the individual level in South Africa. As such, efforts to improve the quality of life and, particularly, the well-being of South Africans must take a general (for example, promote socioeconomic well-being) and group-specific approach.

### Key findings

1. A measure of well-being must be revisited and adjusted when measuring well-being in multicultural settings.
2. Individual SES (income and education) is a vital determinant of well-being.
3. Public health research must adopt a more sociological approach to improve the accuracy and implementability of findings when using standardised measures of well-being.
4. Personal culture, circumstance and collectiveness are significant for interpreting results from well-being research.

## Data Availability

Data included in this report are available on request through the corresponding author (Adekunle Adedeji).
